# Agricultural diversification and intra-household dietary diversity: Panel data analysis of farm households in Bangladesh

**DOI:** 10.1371/journal.pone.0287321

**Published:** 2023-06-23

**Authors:** Mohammad Jahangir Alam, Ismat Ara Begum, Tamanna Mastura, Avinash Kishore, Jim Woodhill, Kuhu Chatterjee, Tamara Jackson

**Affiliations:** 1 Department of Agribusiness and Marketing, Bangladesh Agricultural University, Mymensingh, Bangladesh; 2 Department of Agricultural Economics, Bangladesh Agricultural University, Mymensingh, Bangladesh; 3 International Food Policy Research Institute, New Delhi, India; 4 Environmental Change Institute, University of Oxford, Oxford, United Kingdom; 5 The University of Adelaide, Adelaide, South Australia; MRC Unit The Gambia at LSHTM, GAMBIA

## Abstract

This paper investigates the associations between agricultural diversification and dietary diversity among men, women and children of farm households in Bangladesh. Using three waves of nationally representative Bangladesh Integrated Household Survey (BIHS- 2011–12, 2015 and 2018) data and a panel data model, the study found that agricultural production diversification can emphasize the dietary diversification across the life cycle of farm household members. The balanced panel data reveals that agricultural production diversification has a statistically significant and positive impact on dietary diversity of individual members (men, women and children) of farm households. Moreover, other important factors that impact on agricultural diversification and improve dietary diversity like women’s education and employment, commercialization of farm households, access to non-farm income sources, and access to information facilities also have a strong association in improving dietary status, food and nutritional security as well. Although there is currently limited diversity in the diets of adult men, women and children of rural farm households, the situation can be improved over time with proper and consistent support. The findings suggest policy interventions should target not only agricultural diversification but also improve women’s education and on and off farm employment opportunities and facilitate better access to information for rural households. These recommendations can support improved dietary diversity for all household members under different settings.

## Introduction

Almost 815 million people are chronically malnourished around the world [1]. The majority of these people are living in low and middle-income nations. Though inadequate food consumption is a significant contributor to malnutrition or undernutrition, simply having access to an adequate supply of food is not sufficient to ensure adequate levels of the nutrients necessary for good health [1, 2]. As a result, eating a variety of foods is vital for avoiding micronutrient deficits [3, 4]. The prevalence and severity of malnutrition have far-reaching effects on both the economic and social progress of a country [5]. Malnutrition is a worldwide problem regardless of age, gender, race, or socioeconomic status, but the rate is higher among children in rural settings than their urban counterparts [6, 7]. The causes of malnutrition in rural areas are diverse and complex, but some of the more likely causes include low incomes, limited access to markets, and inadequate educational experiences [8, 9]. Climate change, natural disasters, and other environmental factors may also play a role. Nutritional deficiency may also be a result of gender disparities. In South Asia, the Gender Inequality Index (GII) performs better than Gross Domestic Product (GDP) in predicting low birth weight, suggesting that inequality is a more important determinant of nutrition than poverty [7].

In the context of Bangladesh, 33 million individuals were food insecure in 2010 (USDA, 2010), and by 2020, that population had passed 50 million [10]. The country has made great strides in lowering poverty and hunger in recent times, but several indicators of food insecurity and malnutrition are still challenging [11–13], as more than half of the population are suffering from various forms of malnutrition, particularly the vulnerable populations such as women and children [14]. Agriculture accounts for 13% of Bangladesh’s GDP on average [15], employs over 44% of the workforce and is responsible for the livelihoods of around 77% of the entire population [16]. As a result, agriculture can play a critical role in alleviating malnutrition in Bangladesh. Micronutrient deficiencies are a worldwide problem, particularly in low-income households who eat only one or two staple cereals with sporadic consumption of additional nutrient-dense foods such fruits, vegetables, and animal source protein.

Micronutrient deficits could also be linked to a lack of diversification. Diversification of agriculture is part of economic growth. This process involves a gradual transition from subsistence crops to a diversified, demand-based production system through fast technological change, improved rural infrastructure for agricultural production, and changing consumption patterns [17, 18]. The benefits of agricultural diversification include increased income for farmers, improved food security, and reduced vulnerability to climate change. Agricultural diversification can improve nutritional status in two ways: by diversifying and potentially increasing the horizon of production that can be consumed by a household; and by potentially increasing household income through the diversity in production that is marketable. Other pathways of influence between agriculture and nutrition include agricultural policies which influence food prices; agricultural income spent on health, education and welfare; and women’s empowerment [19]. Diversified agricultural production and diverse diets have been shown to have a positive relationship as greater production diversification results in greater dietary diversity [20–27], although it is not always seen as an effective solution for improving the nutrition of farming households [28, 29].Hence it is important to understand context specific settings and interrelated factors that influence the nutrition status of households and their individual members.

Women, who typically farm with family or on their own, play a crucial role in shaping their families’ diets through agriculture [30]. Despite their significant contributions, they often face gender-based discrimination and limited access to resources. Due to gender inequality, women and children are most vulnerable to food and nutrition security within rural households [31–34], a pattern that is more prominent in South Asia than elsewhere [35]. This is due to the fact that children are reliant on their caregivers for food and nutrition, while women often have limited access to resources and decision-making power. According to a large body of research, diverse diets are also connected with better child nutrition outcomes [3, 36–42]. Studies have noted that a greater dietary diversification score among women of childbearing age is linked to a lower risk of maternal micronutrient deficit, better birth outcomes, and a higher dietary diversity score among their children [3, 4, 43, 44]. The majority of previous research has relied on the household dietary diversity score (HDDS) to quantify the variety of foods consumed by the members within the families. HDDS can be helpful in identifying potential dietary alternatives for family members [25], but food distribution throughout the household is not necessarily fair [45]. A review of existing studies has emphasized the need to examine individual dietary diversity status to understand the true impacts for different household members [46, 47]. Thus, following these suggestions, this study investigates the present scenario in Bangladesh. It aims to contribute to the existing literature by examining the relationship between agricultural diversification and intra-household dietary diversity among farm households by using panel data analysis, after controlling for other factors that may influence food consumption patterns of men, women and children across their life span. This research focused on farm households, as they are likely to have a higher degree of engagement in agricultural activities and therefore, may experience different impacts of agricultural diversification on dietary diversity compared to non-farm households. By examining the relationship between agricultural diversification and intra-household dietary diversity, this study can inform policy decisions aimed at promoting agricultural diversification as a means of improving gender-sensitive nutrition outcomes at the household level.

The remainder of the paper is organised as follows: the Materials and methods section presents the methodology followed by the Results and Discussion sections. Finally, the paper ends with a brief conclusion.

## Materials and methods

### Data

This research made use of Bangladesh Integrated Household Survey (BIHS) data collected in Bangladesh in 2012, 2015, and 2018. The International Food Policy Research Institute (IFPRI) surveyed total of 6,503 households from 325 villages. Among these 6,503 households, 4,423, 4,619 and 4,886 households were “Nationally Representative (representative of Rural Bangladesh)” respectively in 2012, 2015 and 2018 at the division level (Barisal, Chittagong, Dhaka, Khulna, Rajshahi, Rangpur, and Sylhet), and the remaining households fall under a different stratum referred to as the “Feed the Future Zone (FTF)”. FTF is a whole-of-government initiative led by USAID, aiming to improve the livelihood and nutritional status of households globally, and Bangladesh is one of the targeted countries. The target region in Bangladesh is the south and southwest region of the country. Households under the FTF zone have also been considered in this study.

Households which have never been involved in agricultural production (crop production, livestock rearing, and fisheries cultivation) have not been included in this study, as agricultural diversification in the case of these households cannot be measured. To achieve a balanced panel dataset with the common households who have participated in these surveys in all three waves, households which have split up during the time period of 2012–2018 have been excluded (Table 1). Consequently, our sample size is less than that of the official BIHS data. The final sample is a balanced panel of total 12,279 individuals in each round from the 4,093 group of farm-households with complete survey information. Among them a balanced panel of total 3,512, 4,082 and 3,436 group of farm-households have been used with men, women and child members.

**Table 1:** Sample description.

Items	Wave 1 (2012)	Wave 2 (2015)	Wave 3 (2018)	Panel (Balanced)
No. of households in BIHS	6,503	6,437	5,605	
Farm households	4,499	4,617	4,892	4,093
Number of households with men	3,864	3,883	4,075	3,512
Number of households with women	4,479	4,560	4,816	4,082
Number of households with children	3,706	3,770	3,827	3,436

Source: Authors’ estimation (BIHS 2012, 2015 and 2018)

The BIHS survey data contains information on agricultural production (crop, fisheries and livestock); on harvest at the household level; household characteristics, e.g., assets, income; and consumption information for about 299 food items for the seven days prior to the survey. We used the rich information in this data set to analyze the linkages between our main outcome variables—agricultural production diversity and individual dietary diversity.

### Measurement of agricultural diversification

Farmers who adjust their specialization from one crop (or livestock, or fisheries) to a more varied portfolio of crops, livestock, and fisheries are said to have diversified their agriculture system. This shift towards high-value agriculture is of particular interest to us in this study, as it provides a novel strategy for boosting farm incomes beyond the traditional methods of raising crop yields, acreage under cultivation, and levels of agricultural production [48–51].

The main explanatory variable of this study is the Production Diversification Score (PDS), which is simply a count of all the food crops, fish and livestock products produced by a household. The simple count makes it possible to combine crops and animal products produced by the household in the scores [29, 52–59]. This score is calculated by excluding the number of non-food crops from the total value of crop, fish and livestock counted. The PDS does not take into account the proportions of land allotted to each crop, as a measure of diversity, because other types of production like livestock, fish, and dairy are added to the mix. For instance, poultry and dairy can be raised even if farmers don’t have any suitable area to cultivate.

### Measurement of dietary diversity

According to the FAO (2013), dietary diversity is a proxy for nutrient adequacy of the diet and a qualitative measure of food consumption that indicates household access to a variety of foods. Dietary diversity scores, as detailed in the guidelines, are derived from a direct score of the number of different food categories consumed by a family or a person during a specified period. Two commonly used recall periods for collecting dietary data are the 24-hour recall period and the 7-day recall period. Each recall period has its own mix of benefits and drawbacks when used to calculate the dietary diversity score. The 24-hour recall period can capture detailed information on the types and quantities of food consumed by the individual and is less prone to recall bias since the individual is asked to recall their food intake only for the previous 24 hours. But it may not reflect the seasonal or cultural variation in the diet or variation in diet over time. On the other hand, the 7-days recall period may capture the seasonal or cultural variation in dietary intake over a longer period, providing a more comprehensive view of an individual’s diet and dietary pattern. But it is more prone to recall bias since the individual is asked to recall their food intake for the previous 7 days, which is more difficult to accurately recall the food intake over a longer period. Thus, the choice of recall period depends on the research question, the study design, and the available resources. In the case of our study, we have used secondary datasets that use individual dietary data assembled according to the 24-hour recall period.

The goal of calculating an individual’s dietary diversity score is to indicate the degree to which their diet provides an adequate amount of essential nutrients. In order to assess whether or not a person’s diet is adequate in terms of macro and/or micronutrients, dietary diversity scores have been validated for a variety of age and sex groups. Scores have been positively correlated with adequate micronutrient density of complementary foods for infants and young children, and macronutrient adequacy of the diet for non-breast-fed children [4, 40, 42, 60], adolescents [61] and adults [3, 62, 63].

A person’s diet quality can be measured using the Individual Dietary Diversity Score (IDDS), which has been logically established [64]. By counting the number of food groups individually consumed by men, women and children out of 9 food groups in the last 24 hours prior to the survey, we have calculated the Men’s Dietary Diversity Score (MDDS), the Women’s Dietary Diversity Score (WDDS), and the Children’s Dietary Diversity Score (CDDS) [3, 16]. The following nine food groups are used to calculate the IDDS of men, women and children: staples, leafy vegetables, vitamin A rich vegetables and fruits, other vegetables and fruits, meat and fish, organ meat, egg, pulses and nuts; milk and milk products. The value of IDDS varies from 0 to 9; 9 means maximum diversity and 0 means no diversity. Each food group counts towards the individual score if a food item from the group is consumed by anyone in the household in the previous 24h recall period [65].

### Econometric model

We have three waves of BIHS survey data for households of rural Bangladesh. We used these data to analyze the impact of the independent variables on the dependent variable. Our dependent variable, dietary diversity (DD_**i**_), is a count variable that can take a range of values and is not normally distributed. So, we used the Poisson model for our analysis [66, 67]. As we have intra-household data on food consumption, the nature of the data also suggests us to follow a seemingly unrelated regression (SUR) model. We have trialed both of the models and found that the distribution of data and fitness of model logically fulfill the properties of a Poisson regression model and also the results from the Poisson regression have been found to be reliable with the expected outcome of this study compared to that of the SUR model.

We have analyzed a set of balanced panel data to determine the impact of independent variables on the dependent variable by following a Fixed Effects model specification, which was selected based on the findings from the Hausman test and Likelihood Ratio test. In the panel data, we have repeated observations of the same individuals or groups over time. In such data, there may be individual-level factors that remain constant over time and affect the outcome variable of interest, but are unobserved or unmeasured in the data. If these unobserved individual-level factors are correlated with the explanatory variables of interest, the regression coefficients estimated using pooled regression or random effects models will be biased and inconsistent. By including fixed effects in the regression model, we can control for such unobserved individual-level heterogeneity, because fixed effects capture the variation in the outcome variable within individuals over time. In other words, fixed effects control for all time-invariant individual-level factors, and only estimate the effect of time-varying factors on the outcome variable. This makes the fixed effects estimator unbiased and consistent.

To analyze the relationship between agricultural diversification and dietary diversity this study has used regression models of the following form:

$${DD}_{i}=\beta_{0}+\beta_{1}{AD}_{i}+\beta_{2}{MA}_{i}+\beta_{3}X_{i}+u_{i}$$

Where;

DD_i_ = Dietary Diversity of individual members in household i (MDDS, WDDS, CDDS)

Men’s Dietary Diversity Score (MDDS) = Number of food groups consumed by the adult male members (age > = 18 years) of the household in the last 24h.

Women’s Dietary Diversity Score (WDDS) = Number of food groups consumed by the adult female members (age > = 18 years) of the household in the last 24h.

Children’s Dietary Diversity Score (CDDS) = Number of food groups consumed by the child members (age < 18 years) of the household in the last 24h.

AD = Agricultural Diversification of household i (PDS)

Production Diversification Score (PDS) = Number of the different food crops and animal products produced by a household.

MA_i_ = Vector of market access indicator of household i such as distance to the closest market and the availability of non-farm income sources for household i.

X_i_ = Vector of other household characteristics, such as the age of household head, household size, education of household head etc.

μ_i_ = error term.

With the Poisson distribution, it is assumed that a positive and significant estimate for β_1_ implies that higher production diversity is associated with higher dietary diversity. Specifically, an estimate of a coefficient specifies the percentage change in the dietary diversity score for every one unit change in the explanatory variable.

## Results

### Descriptive statistics

The primary outcome variables of our study are the dietary diversity scores of men, women and children, which are also a proxy measure of representing the intra-household dietary status of the members of farm households. The main explanatory variable is the production diversification score of each farm household. The other control variables are farm size, market distance, market participation, non-farm income, annual income, access to information and other socio-demographic characteristics (see S1 Table). We start the analyses by firstly summarizing the main outcome variables of measuring dietary diversity of the individual members across the farm households. There have been significant changes in the dietary diversity scores of men, women and children over the years.

Initially, we performed parametric tests to compare the means of outcome variables in order to characterize descriptive statistics over three waves of the panel. From the table in S2 Table, it is evident that MDDS, WDDS and CDDS increased from the first wave to second wave but decreased from the second to third wave. The average DDS for adult men was lowest in 2012 (2.778) and increased gradually over the next two years to reach 3.002 in 2018 and these changes were statistically significant at 1% level of significance. The dietary diversification of women and children were lower compared to the dietary diversification of adult male members within the farm households in the year 2012, which gradually increased in the next two years. The average DDS for adult women in the overall sample was 2.997 with an SD of 0.833. The mean DDS for adult women was lowest in 2012 (2.764) and increased gradually over the next two years to reach 3.037 in 2018. These changes were also statistically significant at 1% level of significance. The average DDS score for the children in the study was 2.999, with a standard deviation of 0.878. In 2012, the mean DDS for children was 2.745, and it has increased consistently with a 1% level of significance over the past six years, reaching 3.052 in 2018. However, the findings indicate an overall improvement in dietary diversity among adult men, adult women, and children over time.

The findings presented in the S3 Table describe the mean and standard deviation of different explanatory variables and the mean differences for three different periods: 2012–2015, 2015–2018, and 2012–2018. The table also shows the pooled means for each variable for all three periods. From the results, we have found that the main explanatory variable PDS slightly decreases from the first wave to the second wave but again increases from the second to the third wave. The mean difference of PDS between 2012 and 2015 is -0.377, between 2015 and 2018 is 0.754, and between 2012 and 2018 is 1.131. All these changes are statistically significant at the 1% level. These findings suggest that the PDS has improved over the years and has had a significant impact on the dietary diversity of individual household members in rural households. Among the farm households, the mean difference for farm size shows a positive value for the 2015–2018 period, indicating an increase in the average farm size during this period. However, the mean differences for the 2012–2015 and 2012–2018 periods show negative values, indicating a decrease in the average farm size during these periods. But the market participation of the farms increases with each wave, though the changes are found to be insignificant. The mean difference for market participation shows a positive value for both the 2012–2015 and 2012–2018 periods, indicating an increase in market participation during these periods. Again, the mean difference for 2015–2018 shows a negative value, indicating a decrease in market participation during this period. However, the mean difference for non-farm income shows positive values for all three periods, indicating an increase in the income from non-farm activities in the farm households during all three periods. Moreover, the earning status of women in households significantly increases over time, and these changes may support women’s empowerment.

Considering the household characteristics, results highlighted that the mean differences for sex and age of the household head, age and education of adult women, household size, share of children and elders, and access to information have consistent positive or negative values for all three periods, indicating a consistent increase or decrease in the mean values of these variables over time. Overall, the findings in the table suggest that there have been changes in the mean values of different variables over time, and these changes vary depending on the specific variable and the time period under consideration.

Table 2 displays the percentage and frequency of various food groups consumed over the previous 24 hours by men, women, and children in different waves. Staples which include rice, wheat, maize, potato, sweet potato, bread etc. are the most frequently consumed food group among all age and gender groups, with a consumption percentage of nearly 100%. The second most consumed food group is fish and meat which is found to be consumed by over 80% of the respondents. However, the consumption of different types of fish and meat (beef, mutton, chicken etc.) is higher among men than women and children. On the other hand, the consumption of egg and organ meats like- liver, kidney, heart, brain etc. is found negligible across all groups. The third highest consumed food group is leafy vegetables (spinach, amaranth, lettuce, and other green leafy vegetables) which are the most frequently consumed among all vegetable groups, and the consumption percentage is highest among children. Though the consumption percentage of Vit-A rich vegetables and fruits (carrots, pumpkin, sweet potato, squash, papaya, mango, and other orange and yellow fruits and vegetables) is relatively low across all age and gender groups, other vegetables and fruits (tomato, onion, okra, eggplant, cucumber, green beans, banana, apple, guava, and other fruits and vegetables etc.) are consumed more by women and children than men. Then, the consumption percentage of pulses and nuts is the lowest among all food groups, with a range of 5.98% to 7.49%. Lastly, the consumption of milk and milk products like yogurt, cheese etc. is lower in men than women and children, with the highest consumption percentage in children.

**Table 2:** Summary statistics of food consumption among men, women and children.

Food groups	Wave	Men		Women		Children	
		Freq.	Percent.	Freq.	Percent.	Freq.	Percent.
Staples	Pooled	10,284	99.90	12,148	99.91	9,941	99.73
	2012	3,478	99.91	4,074	99.95	3,362	99.67
	2015	3,399	99.85	4,042	99.98	3,341	99.70
	2018	3,407	99.94	4,032	99.80	3,238	99.82
Leafy vegetables	Pooled	6,244	60.66	7,353	60.47	5,912	59.31
	2012	1,934	55.56	2,270	55.69	1,787	52.98
	2015	2,249	66.07	2,687	66.46	2,174	64.88
	2018	2,061	60.46	2,396	59.31	1,951	60.14
Vit-A rich vegetables and fruits	Pooled	242	2.35	383	3.15	342	3.43
	2012	2	0.06	8	0.20	9	0.27
	2015	203	5.96	308	7.62	283	8.45
	2018	37	1.09	67	1.66	50	1.54
Other vegetables and fruits	Pooled	3,266	31.73	4,277	35.18	3,740	37.52
	2012	434	12.47	554	13.59	555	16.45
	2015	1,527	44.86	1,977	48.90	1,701	50.76
	2018	1,305	38.28	1,746	43.22	1,484	45.75
Fish and meat	Pooled	8,905	86.51	10,423	85.72	8,378	84.05
	2012	3,013	86.56	3,483	85.45	2,809	83.28
	2015	2,939	86.34	3,457	85.51	2,825	84.30
	2018	2,953	86.62	3,483	86.21	2,744	84.59
Organ meat	Pooled	0	0	0	0	0	0
	2012	0	0	0	0	0	0
	2015	0	0	0	0	0	0
	2018	0	0	0	0	0	0
Egg	Pooled	10	0.10	8	0.07	22	0.22
	2012	10	0.29	8	0.20	22	0.65
	2015	0	0	0	0	0	0
	2018	0	0	0	0	0	0
Pulses and nuts	Pooled	616	5.98	747	6.14	747	7.49
	2012	498	14.31	572	14.03	493	14.62
	2015	64	1.88	77	1.90	127	3.79
	2018	54	1.58	98	2.43	127	3.91
Milk and milk products	Pooled	1,476	14.34	1,631	13.41	2,019	20.25
	2012	323	9.28	329	8.07	430	12.75
	2015	647	19.01	750	18.55	913	27.25
	2018	506	14.84	552	13.66	676	20.84

### Econometric model

In this study, panel data Poisson regression models have been used to analyze the association between agricultural diversification and intra-household dietary diversity within the farm households. This study has chosen the PDS to use as explanatory variables for measuring the impact of agricultural diversification on MDDS, WDDS and CDDS of rural farm households. There is a possibility that the production diversification may connect with some of the factors that were left out of the estimation and could result in skewed findings. These factors are mainly socioeconomic and demographic factors that are closely related to the composition of households. So, to test for such bias, this study has also checked the robustness of the regression models. The final results are shown in the following tables.

Table 3 shows the pooled data regression results with coefficients and standard errors. The coefficients represent the change in the dependent variable associated with a one-unit increase in the corresponding independent variable. The standard errors are used to test the statistical significance of the coefficients. The model is used to analyze the impact of production diversification on the dietary diversity of men, women, and children using pooled data from three time periods (2012, 2015, and 2018). In general, the results indicate that production diversification of farm households has a positive and highly significant relationship with the dietary diversity scores of men, women and children. In the pooled specification, we observe that an increase in the number of food species produced is associated with a 0.3% increase in the MDDS and 0.4% increase in both the WDDS and CDDS. The model includes several control variables such as non-farm income, market distance, and market participation, earning status of women, access to information and other demographic characteristics. We found that non-farm income has a positive but insignificant effect on MDDS and WDDS, while it has a significant positive effect on CDDS. Market distance which is the physical distance from the farm household to the nearest market, has a negative but insignificant effect on MDDS and WDDS, while it has a negative and significant effect on CDDS. However, market participation is the proxy measure of the commercial nature of farm households, has a significant positive effect on all three dietary diversity scores. On the other hand, earning status of women has a significant positive effect on MDDS and CDDS, but an insignificant effect on WDDS, though women’s education has a significant positive effect on all three scores.

**Table 3:** Results of production diversification on men, women and children dietary diversity (Pooled Poisson model).

Variables	MDDS	WDDS	CDDS
PDS	0.003*** (0.001)	0.004*** (0.001)	0.004*** (0.001)
Non-farm income	3.60e-08 (2.50e-08)	4.26e-08 (2.59e-08)	5.57e-08** (2.77e-08)
Market distance	-0.001 (0.001)	-0.002* (0.001)	-0.001 (0.001)
Market participation	2.675e-04*** (1.025e-04)	2.997e-04*** (9.55e-05)	2.709e-04** (1.120e-04)
Earning status women	0.011* (0.006)	0.007 (0.006)	0.019*** (0.007)
HH head sex	-0.003 (0.013)	0.010 (0.007)	0.008 (0.009)
HH head age	0.001*** (2.345e-04)	0.001*** (2.202e-04)	0.001*** (2.824e-04)
HH head education	0.003*** (0.001)	0.004*** (0.001)	0.003*** (0.001)
Women education	0.004*** (0.001)	0.004*** (0.001)	0.004*** (0.001)
HH size	0.009*** (0.002)	0.008*** (0.002)	-0.001 (0.002)
Share of children	-0.001*** (1.713e-04)	-0.001*** (1.543e-04)	4.744e-04** (2.380e-04)
Share of elders	-0.001*** (1.842e-04)	-0.001*** (1.647e-04)	-7.62e-05 (2.654e-04)
Farm size	9.11e-05*** (2.52e-05)	6.28e-05*** (2.37e-05)	1.013e-04*** (2.79e-05)
Access to information	0.048*** (0.009)	0.062*** (0.008)	0.065*** (0.010)
Year dummy 1 (base 2012)	0.113*** (0.007)	0.133*** (0.006)	0.145*** (0.007)
Year dummy 2 (base 2012)	0.050*** (0.008)	0.071*** (0.007)	0.084*** (0.008)
Constant	0.842*** (0.020)	0.838*** (0.016)	0.798*** (0.022)
Log likelihood	-16244.45	-19243.061	-15891.601
Wald χ2	865.36	1292.03	910.36
Observations	10,294	12,159	9,968

Notes: Robust standard errors are in the parentheses; *** p<0.01, ** p<0.05, * p<0.1

Based on the results of the other factors, it appears that household head sex and age have an insignificant effect on all three dietary diversity scores, while household head education has a significant positive effect on all three scores. The impact of household head’s education and women’s education on individual dietary diversity are also significant and positive. Household size is a crucial factor to determine the diet quality of household members and the results found that this variable has a significant positive effect on MDDS and WDDS, but an insignificant effect on CDDS. The share of children has a negative and significant effect on MDDS and WDDS, but a positive and significant effect on CDDS. The share of elders has a negative and significant effect on MDDS and WDDS, but an insignificant effect on CDDS. Farm size usually represents the capacity of farming activities of farmers and has a significant positive effect on dietary diversity scores of all the members of farm household. Finally, access to information has a significant positive effect on all three scores.

Additionally, the model includes year dummies to control for unobserved time-varying factors. In this study, the year dummies indicate a significant positive trend over time for all three dietary diversity scores of men, women and children of farm households. The model has good explanatory power, as indicated by the Wald χ2 statistics, which are all statistically significant at the 1% level. The log likelihood values indicate that the model fits the data well. Overall, the results supported that agricultural production diversification and several other factors have a significant impact on intra-household dietary diversity of farm households in Bangladesh.

Since the fixed-effect regression model is an important tool in panel data analysis to obtain accurate estimates of the relationship between the outcome variable and explanatory variables of interest, this study tried to estimate the fixed-effect regression model (Table 4) to control for unobserved individual-level heterogeneity that could change the estimates of the regression coefficients.

**Table 4:** Results of production diversification on men, women and children dietary diversity (fixed-effect Poisson model).

Variables	MDDS	WDDS	CDDS
PDS	0.001 (0.001)	0.003*** (0.001)	0.004*** (0.001)
Non-farm income	1.69e-08 (3.29e-08)	1.73e-08 (3.63e-08)	2.22e-08 (4.47e-08)
Market distance	-3.738e-04 (0.001)	-0.001 (0.001)	-2.048e-04 (9.582e-04)
Market participation	2.004e-04 (1.491e-04)	1.305e-04 (1.363e-04)	7.43e-05 (1.696e-04)
Earning status women	0.015* (0.008)	0.019** (0.007)	0.038*** (0.009)
HH head sex	-0.031 (0.027)	0.007 (0.014)	0.019 (0.019)
HH head age	0.001 (0.001)	0.001 (0.001)	0.001 (0.001)
HH head education	-2.260e-04 (3.207e-03)	0.002 (0.002)	-0.001 (0.003)
Women education	5.445e-03** (0.003)	0.007*** (0.003)	0.006 (0.004)
HH size	0.005** (0.003)	0.001 (0.004)	-0.020*** (0.005)
Share of children	0.003 (0.004)	-2.363e-04 (2.674e-04)	0.001*** (4.021e-04)
Share of elders	-0.001*** (3.161e-04)	-0.001** (2.747e-04)	4.026e-04 (4.299e-04)
Farm size	-0.001*** (4.56e-05)	1.225e-04*** (4.09e-05)	1.186e-04** (4.92e-05)
Access to information	0.029** (0.013)	0.027** (0.011)	0.023* (0.013)
Year dummy 1 (base 2012)	0.121*** (0.008)	0.140*** (0.007)	0.162*** (0.008)
Year dummy 2 (base 2012)	0.067*** (0.011)	0.086*** (0.010)	0.123*** (0.012)
Log likelihood	-8403.4796	-10506.948	-8068.1448
Wald χ2	441.97	725.29	632.64
Observations	10,294	12,159	9,968
Number of HH	3,512	4,082	3,436

Notes: Robust standard errors are in the parentheses; *** p<0.01, ** p<0.05, * p<0.1

In the fixed effect specification, the production diversification, which measures the number of different agricultural produces (crops and animal species) produced by each household, has a positive impact on increasing the dietary diversity of all three groups. This indicates that households with more diversified production systems have higher dietary diversity scores. More specifically, the coefficient for PDS is statistically significant only for women and children in the farm households, whereas the coefficient for men is positive but not statistically significant. Results found that an increase in the number of food species produced is associated with a 0.3% increase in the WDDS and 0.4% increase in the CDDS.

This model also includes a range of other covariates. It has been found that non-farm income, market distance, and market participation all have positive effects on WDDS and CDDS but the effects are not conclusive. This means that income from sources other than farming, access to markets, and selling farm products on the market can all help improve dietary diversity. However, household head sex and education are found to have varying effects on the different dietary diversity scores. The coefficient for educational qualification of household head variable is positive and statistically significant for all three groups, indicating that older household heads are associated with higher dietary diversity scores. Considering the household size, results found that the coefficient of this variable is positively associated with the dietary diversity of men and women but has a negative association with the dietary diversity of children. This indicates that larger households are associated with lower dietary diversity scores for children. On the other hand, an increase in the number of children in household size may have positive and significant impacts on the diet diversity of those children but may lower the diet diversity of adult women of those households. An increase in the share of elderly members can also significantly decrease the diet diversity of adult men and women. The impact of household women’s education on individual dietary diversity are found to be significant and positive. Also, households with female members who are engaged in earning from farm and non-farm sources contribute to higher dietary diversity in individual levels, especially for their children. The result also found that ownership of technologies such as television, radio and mobile phone which provide access to information have a strong correlation and significant influence on dietary diversification of men, women and children.

In this fixed-effect analysis time is considered as a variable by including the three dummies from the survey waves using 2012 as the reference. Both the 2015- and 2018-year dummies are highly significant at the individual level for men, women, and children, demonstrating the significance of time. Moreover, the positive coefficient of the dummy variable indicates that diversifying agricultural production can prioritize the improvement of the dietary and nutritional status of farm household members throughout their lifespan.

## Discussion

Diversification of agricultural production can improve food security and the nutritional status of households by increasing the availability and accessibility of nutrient-rich foods and reducing the risk of failure. It can also provide additional income streams for farmers, improving their economic well-being [21, 52, 68–73]. This study tried to draw out the impact of agricultural diversity on the dietary diversity of individual household members within farm households in Bangladesh.

Our results provide empirical evidence of the positive association between agricultural production diversification and intra-household dietary diversity using panel data. Results found that the impact of agricultural production diversification is higher among women and children within farm households than among men [21, 74–76]. In other words, when farm households diversify their agricultural production by growing a wider range of crops or raising different types of livestock, it has a greater impact on improving the dietary diversity of women and children in those households compared to men. This finding indicates that women and children may have a higher dietary sensitivity to agricultural production diversification. It could be due to several factors, such as women being more involved in food preparation and decision-making regarding household nutrition or children having higher nutrient requirements for growth and development. For example, in a rural farming community in Kenya, households that diversified their crop production by growing different types of vegetables, fruits, and grains experienced an increase in the dietary diversity of women and children. This led to better health for the women and their children, as they were able to feed their families more nutritious foods [22]. Studies in Nepal and Ethiopia using a similar method, based on nine and seven food groups respectively, also found that production diversity played an important role in improving child and maternal DDS [77, 78]. So, agricultural diversification can be an effective way to improve the well-being of women and children in rural areas.

We also found significant association between species count and dietary quality, suggesting that to add one more food group into a diet, a farm household may need to diversify the production by about 25–30 species. In Tanzania and Sub-Saharan Africa, farmers were suggested to diversify their production by nine or more species to increase dietary diversity by one more food group [52, 79]. The marginal ratio suggested in our result may not be practical for all households or regions, but it is possible to promote the year round cultivation of diverse crops or livestock that are suitable for the local context. So, farm households need to be encouraged to diversify their agricultural production by providing training, technical support, and financial incentives to increase the availability and accessibility of nutrient-rich foods and reduce the risk of failure.

This study also found that dietary diversity among household members, especially women and children, can be positively impacted by non-farm income, proximity to markets, and market participation in selling agricultural products [21, 80, 81]. In general, non-farm income refers to income sources other than agricultural activities, such as off-farm employment or entrepreneurial ventures. When households have additional sources of income beyond farming, it can positively affect their dietary diversity as this additional income allows households to have more financial resources to purchase a wider range of foods, including more diverse and nutritious options. For example, a study conducted in rural Nigeria found that households with non-farm income were more likely to consume a diverse range of fruits and vegetables, as well as animal-source foods like meat and dairy. This was due to their increased purchasing power and ability to afford these more expensive food items [82]. In addition, households with non-farm income are also more likely to invest in improved agricultural techniques and inputs, such as fertilizer and irrigation systems, which results in increased productivity and higher crop yields [83, 84]. On the other hand, proximity to markets plays a role in improving dietary diversity by providing greater access to a variety of food products. When households are located near markets, they have easier access to a wider range of food items, including fresh produce, meat, dairy, and other nutritious foods. This accessibility to diverse food options enhances the ability of household members, especially women, and children, to consume a more varied and balanced diet [28, 85]. Similarly, market participation in selling agricultural products also contributes to improved dietary diversity. When households actively engage in selling their agricultural produce in local markets, they not only generate income but also have exposure to a broader range of food choices available in those markets. This exposure can influence household members’ dietary patterns by introducing them to new foods and encouraging the consumption of locally available nutrient-rich foods [28, 86, 87]. So, all these evidences underscore the need of boosting access to markets and diversifying revenue sources for farm households in ensuring food and nutrition security because many women and children, particularly in low-income countries, may have limited access to diverse foods due to factors such as poverty, limited food availability, and cultural practices. Diversifying income sources and participating in markets selling agricultural produce are both strategies which can help rural families earn more income and therefore purchase a wider range of foods. Greater accessibility to markets can expand the availability of diverse foods to improve the quality and diversity of their diets, as well as increase their food security [88–92]. Considering the importance of these factors, investment in infrastructure, transportation networks, and market facilities in rural areas are suggested, which will enhance the accessibility of markets for rural households, reducing transportation costs and time.

In South Asia, the earning status of women is largely associated with child nutrition. Previous studies have found a positive and significant relationship between the earning status of women and the dietary diversity status of the farm-household, indicating that women’s empowerment has a significant impact on improving diet and nutritional status [21, 75, 78, 93]. Similarly in Bangladesh, women are crucial actors in the food system, and their empowerment promotes dietary diversity as well as household food security [21, 74]. Our research also supports the idea that when farm women have more education and become financially independent, it helps their families be more food secure. In the case of our study, we found that women’s education has a significant positive effect on child dietary diversity in the pooled model. The effect was found insignificant but positive under the fixed-effect condition, which is also acceptable because there may be other factors that have a stronger influence on child dietary diversity. For example, we found that the earning status of women has a highly significant influence on child dietary diversity in the households. However, women’s education may not necessarily lead to improved dietary practices in the household, as women may have limited decision-making power within the household or face social and cultural barriers that prevent them from making changes to dietary practices. This finding highlights the importance of investing both in education and economic opportunities for women in rural areas, not only for their own empowerment but also for the wellbeing of their families and communities. It is also suggested that programs should promote gender equality, support women’s leadership, and provide opportunities for women to actively participate in decision-making processes related to agriculture and nutrition.

Moreover, results showed that access to information through television, radio and mobile phones can have a significant influence on dietary diversification of men, women, and children. This is because these media platforms can provide important information on the benefits of consuming a variety of foods, as well as how to prepare them. Additionally, they can also promote the consumption of locally available and affordable nutrient-rich foods. By providing knowledge and awareness of the importance of a diverse diet, these technologies can promote better nutritional outcomes and improve overall health and well-being. Thus, policy efforts should focus on promoting nutrition education and awareness campaigns targeting households, particularly women and caregivers. These campaigns can utilize various communication channels, such as television, radio, and mobile phones, to disseminate information on the importance of diverse diets, food preparation techniques, and the nutritional value of locally available foods. By increasing knowledge and awareness, households can make informed choices and adopt healthier dietary practices.

There are some limitations to our study which provides scope for future research. One limitation of our study is that we relied on a 24-hour recall period to gather dietary data as the BIHS survey dataset contains the dietary information at the individual level for the previous 24-hours. While this method can provide valuable information, it may not accurately reflect an individual’s usual dietary habits. In future research, incorporating longer recall periods or using other methods such as food diaries or dietary diversity index may provide more comprehensive insights. Another limitation is that our study used secondary data which only covers panel data from the years 2012, 2015, and 2018, which may not be representative of the current entire population or provide a complete understanding of the phenomenon being studied. Additionally, there may be other factors like behavioral or cultural practices that were not included enough in our analysis that could have influenced the results. Future research could expand on the time frame and consider other variables to better understand the topic more deeply.

## Conclusion

Due to the prevalence of malnutrition in the poorest rural smallholders whose livelihoods depend on agriculture, there has been a growing effort to encourage nutrition-sensitive agricultural research and programs. Agricultural diversification has been suggested to be an important strategic tool for boosting economic development, alleviating poverty, and strengthening food security in the developing nations.

This study attempted to find out whether agricultural diversification influences dietary diversification of individual members of farm households of Bangladesh using balanced panel data from BIHS in 2012, 2015 and 2018. The findings of this study have important implications for policy makers and development practitioners in Bangladesh and other developing countries. Though results found limited diversity in diets of adult men, women and children in rural farm households, the situation is improving over time. Findings imply that agricultural diversification significantly and positively affects dietary diversity within households for adults and children. Although women and children are often at risk of having lower dietary diversity, we found that through time women and especially children are getting equal access to dietary diversification across a range of farm households. This is evidence that agricultural production diversification can support intra-household dietary diversification of rural farm households. Women’s education and employment status were among the significant aspects explaining men, women, and children’s dietary diversity scores when weighing the possible factors related with dietary diversity. Policymakers and development practitioners should consider these gendered impacts when designing and implementing agricultural programs, which need to be integrated across multiple sectors beyond agriculture.

There are multiple pathways by which agricultural diversification can influence nutrition. Understanding local contexts to best promote the most effective pathways is important. However, overall findings suggest policy interventions that not only target agricultural diversification but also on and off farm employment opportunities, commercialization of farm produce, women’s empowerment, and facilitate better access to market and information should be prioritized to improve dietary diversity and hence nutrition outcomes for all household members.

## Supporting information

S1 Table Description of the outcome variables, explanatory variable and other control variables. (DOCX)

S2 Table Descriptive statistics of outcome variables. (DOCX)

S3 Table Descriptive statistics of explanatory variables. (DOCX)
