# Supplementary material for: Agricultural diversification and intra-household dietary diversity: Panel data analysis of farm households in Bangladesh
Source: PLoS One. 2023 Jun 23;18(6):e0287321. doi: 10.1371/journal.pone.0287321 (PMC10289449; doi:10.1371/journal.pone.0287321)
Supplement: S1 Table — (DOCX) [file pone.0287321.s001.docx]

**S1 Table: Description of the outcome variables, explanatory variable and other control variables**

| **Variables** | **Description** | **Sub-factors** |
| --- | --- | --- |
| **Outcome variables** | | |
| Dietary Diversity Score of adult men (MDDS) | Number of food groups (ranges 0-9) consumed by the adult male members (age >= 18 years) of the household in the last 24h. | Count variable |
| Dietary Diversity Score of adult women (WDDS) | Number of food groups (ranges 0-9) consumed by the adult female members (age >= 18 years) of the household in the last 24h. | Count variable |
| Dietary Diversity Score of children (CDDS) | Number of food groups (ranges 0-9) consumed by the child members (age < 18 years) of the household in the last 24h. | Count variable |
| **Explanatory variable** | | |
| Production diversification score (PDS) | Number of the different food crops and animal products produced by a household. | Continuous variable |
| **Other control variables** | | |
| Sex of HH head | 1=Male and 0=Female | Binary variable |
| Age of HH head | Age of the HH head (years) | Continuous variable |
| Education of HH head | Years of formal schooling for the HH head (years) | Continuous variable |
| Age of adult women | Age of the HH adult women (years) | Continuous variable |
| Education of adult women | Years of formal schooling for the HH head (years) | Continuous variable |
| Earning status of adult women | 1=Yes and 0=No | Binary variable |
| Household size | The number of family members in the household | Continuous variable |
| Share of children | Percentage (0-100) of child members (age < 18 years) in the household size (%) | Discrete variable |
| Share of elders | Percentage (0-100) of elderly members (age >18 years) in the household size (%) | Discrete variable |
| Farm size | Total area of land under cultivation (decimal; 1 decimal = 0.01 acres) | Continuous variable |
| Market distance | Physical distance between the household's residence and the nearest market (km) | Continuous variable |
| Market participation | Percentage (0-100) of value of total agricultural produces which is sold in market during the past 12 months (%) | Discrete variable |
| Non-farm income | Income from non-farm activities in past 12 months (Taka) | Continuous variable |
| Access to information | 1=Yes and 0=No | Binary variable |
