# Supplementary material for: Agricultural diversification and intra-household dietary diversity: Panel data analysis of farm households in Bangladesh
Source: PLoS One. 2023 Jun 23;18(6):e0287321. doi: 10.1371/journal.pone.0287321 (PMC10289449; doi:10.1371/journal.pone.0287321)
Supplement: S2 Table — (DOCX) [file pone.0287321.s002.docx]

**S2 Table:** **Descriptive statistics of outcome variables**

| **Outcome Variables** | **Mean and Standard Deviation (SD)** | | | | **Mean diff. (2012 vs. 2015) &**  **(t value)** | **Mean diff. (2015 vs. 2018) &**  **(t value)** | **Mean diff. (2012 vs. 2018) &**  **(t value)** |
| --- | --- | --- | --- | --- | --- | --- | --- |
|  | **Pooled** | **2012** | **2015** | **2018** |  |  |  |
| Dietary Diversity Score of adult men (MDDS) | 2.975 (0.815) | 2.778 (0.771) | 3.149 (0.840) | 3.002  (0.791) | 0.371***  (19.21) | -0.147***  (-7.58) | 0.214***  (11.60) |
| Dietary Diversity Score of adult women (WDDS) | 2.997  (0.833) | 2.764  (0.768) | 3.191 (0.849) | 3.037  (0.824) | 0.427***  (23.64) | -0.154***  (-8.48) | 0.274***  (15.14) |
| Dietary Diversity Score of children (CDDS) | 2.998 (0.878) | 2.745 (0.810) | 3.200 (0.898) | 3.052  (0.862) | 0.455***  (21.74) | -0.148***  (-7.02) | 0.306***  (14.54) |
| Number of observations (HH group) | 12,279 | 4,093 | 4,093 | 4,093 | - | - | - |

Notes: t-values are in the parentheses; *** and ** indicate significance at 1% and 5%, respectively.
