# Supplementary material for: Agricultural diversification and intra-household dietary diversity: Panel data analysis of farm households in Bangladesh
Source: PLoS One. 2023 Jun 23;18(6):e0287321. doi: 10.1371/journal.pone.0287321 (PMC10289449; doi:10.1371/journal.pone.0287321)
Supplement: S3 Table — (DOCX) [file pone.0287321.s003.docx]

**S3 Table: Descriptive statistics of explanatory variables**

| **Explanatory Variables** | **Mean and Standard Deviation (SD)** | | | | **Mean diff. (2012 vs. 2015)** | **Mean diff. (2015 vs. 2018)** | **Mean diff. (2012 vs. 2018)** |
| --- | --- | --- | --- | --- | --- | --- | --- |
|  | **Pooled** | **2012** | **2015** | **2018** |  |  |  |
| PDS | 8.049  (5.183) | 7.923  (5.586) | 7.546  (4.80) | 8.677  (5.068) | -0.377  (-3.31) | 1.131***  (9.92) | 0.754***  (6.61) |
| Farm size (decimal) | 62.645  (110.485) | 63.885 (110.857) | 64.603 (123.102) | 59.447 (95.750) | 0.719  (0.29) | -5.156*  (-2.11) | -4.437  (-1.82) |
| Market distance (km) | 1.783  (2.654) | 1.744  (1.681) | 1.709 (1.859) | 1.897  (3.852) | -0.035  (-0.60) | 0.188***  (3.21) | 0.153**  (2.60) |
| Market participation (%) | 24.248 (27.241) | 23.729 (26.502) | 24.488 (28.031) | 24.527 (27.167) | 0.759  (1.26) | 0.039  (0.07) | 0.798  (1.33) |
| Non-farm income (Tk.) | 62,801  (96,504) | 41,595 (59,215) | 61,601 (101,969) | 85,208 (114395) | 20005***  (9.54) | 23607***  (11.26) | 43613***  (20.80) |
| Sex of HH head | 0.816  (0.387) | 0.831  (0.375) | 0.818 (0.386) | 0.800  (0.400) | -0.013  (-1.54) | -.0178*  (-2.08) | -0.031***  (-3.63) |
| Age of HH head | 46.06 (13.355) | 43.99  (13.542) | 46.14 (13.291) | 48.04 (12.919) | 2.15***  (7.34) | 1.89***  (6.48) | 4.04***  (13.82) |
| Education of HH head (years) | 3.600  (4.115) | 3.476  (4.090) | 3.599  (4.083) | 3.725  (4.168) | 0.123  (1.35) | 0.127  (1.39) | 0.250**  (2.75) |
| Age of adult women | 38.350 (13.387) | 36.306 (13.019) | 38.344 (13.429) | 40.401 (13.398) | 2.038**  (6.94) | 2.056***  (7.00) | 4.095***  (13.94) |
| Education of adult women (years) | 3.391  (3.616) | 3.227  (3.580) | 3.365 (3.574) | 3.580  (3.683) | 0.139  (1.73) | 0.214**  (2.69 ) | 0.353***  (4.42) |
| Earning status of adult women | 0.768  (0.422) | 0.645  (0.478) | 0.780  (0.415) | 0.879  (0.326) | 0.134***  (14.76) | 0.099***  (10.91) | 0.233***  (25.67) |
| Household size | 4.777  (1.823) | 4.178  (1.505) | 4.772  (1.720) | 5.381  (2.007) | 0.594***  (15.30) | 0.608***  (15.67) | 1.202***  (30.97) |
| Share of children (%) | 35.079 (20.845) | 38.973 (21.525) | 35.529 (20.417) | 30.736 (19.730) | -3.444***  (-7.57) | -4.793***  (-10.54) | -8.237***  (-18.11) |
| Share of elders (%) | 14.493 (20.180) | 6.090  (14.862) | 13.908 (18.840) | 23.481 (22.231) | 7.818***  (18.73) | 9.574***  (22.93) | 17.391***  (41.66) |
| Access to information | 0.886  (0.318) | 0.797  (0.402) | 0.900  (0.300) | 0.960  (0.195) | 0.103***  (15.01) | 0.060***  (8.78) | 0.163***  (23.79) |
| Number of observations (HH group) | 12,279 | 4,093 | 4,093 | 4,093 | - | - | - |

Notes: t-values are in the parentheses; *** and ** indicate significance at 1% and 5%, respectively.
